# Analogies and Differences Between Dental Stem Cells: Focus on Secretome in Combination with Scaffolds in Neurological Disorders

**DOI:** 10.1007/s12015-023-10652-9

**Published:** 2023-11-14

**Authors:** Francesca Santilli, Jessica Fabrizi, Costantino Santacroce, Daniela Caissutti, Zaira Spinello, Niccolò Candelise, Loreto Lancia, Fanny Pulcini, Simona Delle Monache, Vincenzo Mattei

**Affiliations:** 1 Biomedicine and Advanced Technologies Rieti Center, “Sabina Universitas”, Via A.M. Ricci 35/A, 02100 Rieti, Italy; 2 Department of Experimental Medicine, “Sapienza” University, Viale Regina Elena 324, 00161 Rome, Italy; 3 National Center for Drug Research and Evaluation, Istituto Superiore di Sanità, Viale Regina Elena, 29900161 Rome, Italy; 4 Department of Biotechnological and Applied Clinical Sciences, University of L’Aquila, Via Vetoio, 67100 L’Aquila, Italy; 5 Dipartimento di Scienze della Vita, della Salute e delle Professioni Sanitarie, Link Campus University, Via del Casale di San Pio V 44, 00165 Rome, Italy

**Keywords:** Dental stem cells, Conditioned media, Exosomes, Secretome, Scaffolds, Cell therapy

## Abstract

Mesenchymal stem cells (MSCs) are well known for their beneficial effects, differentiation capacity and regenerative potential. Dental-derived MSCs (DSCs) are more easily accessible and have a non-invasive isolation method rather than MSCs isolated from other sources (umbilical cord, bone marrow, and adipose tissue). In addition, DSCs appear to have a relevant neuro-regenerative potential due to their neural crest origin. However, it is now known that the beneficial effects of MSCs depend, at least in part, on their secretome, referring to all the bioactive molecules (neurotrophic factors) released in the conditioned medium (CM) or in the extracellular vesicles (EVs) in particular exosomes (Exos). In this review, we described the similarities and differences between various DSCs. Our focus was on the secretome of DSCs and their applications in cell therapy for neurological disorders. For neuro-regenerative purposes, the secretome of different DSCs has been tested. Among these, the secretome of dental pulp stem cells and stem cells from human exfoliated deciduous teeth have been the most widely studied. Both CM and Exos obtained from DSCs have been shown to promote neurite outgrowth and neuroprotective effects as well as their combination with scaffold materials (to improve their functional integration in the tissue). For these reasons, the secretome obtained from DSCs in combination with scaffold materials may represent a promising tissue engineering approach for neuroprotective and neuro-regenerative treatments.

**Graphical Abstract:**

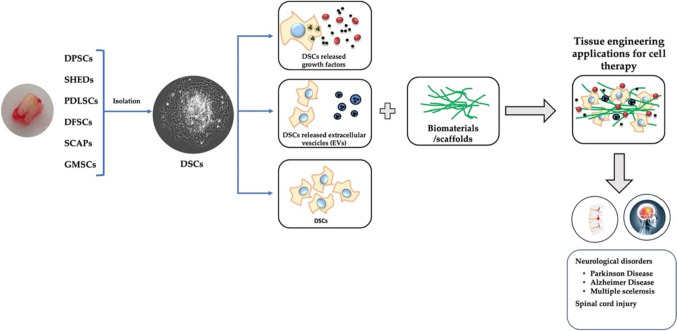

## Introduction

**Fig. 1:**
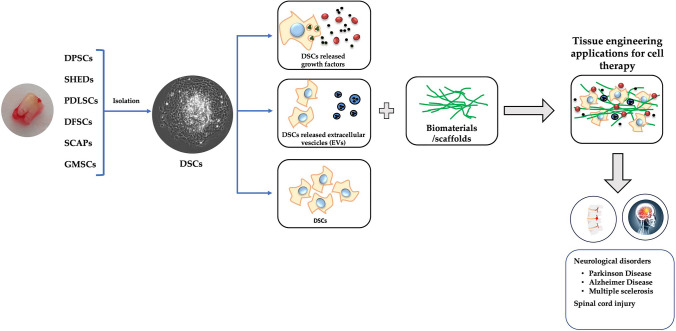
Schematic representation of DSCs in tissue engineering application for CT. DSCs can release growth factors and extracellular vesicles or be used directly with scaffolds for tissue engineering cell therapy in neurological disorders

Stem cells (SCs) are undifferentiated cell populations with high self-renewal capabilities and unlimited differentiation potential [1]. They are found during embryonic development (Embryonic stem cells, ESCs) and in many adult tissues and organs, where they replace dying cells and regenerate damaged tissue. ESCs are pluripotent cells. This means that they can differentiate into cell types belonging to each germ line (ectoderm, mesoderm, and endoderm). Adult stem cells (ASCs), are classified as mesenchymal stem cells (MSCs), Neural stem cells (NSCs), Epithelial stem cells (EpSCs), and Skin stem cells (SSCs) depending on the tissue which they are isolated and have limited differentiation potential. The most studied line of ASCs is the bone marrow-derived line (BM-MSCs), as they were discovered to belong to the hematopoietic niche in the 1970s [2]. However, also BM-MSCs have limited differentiation potential due to their mesodermal origin. This limitation is particularly relevant for the eventual therapeutic application in organs mainly composed of post-mitotic cells, such as the brain. Indeed, although BM-MSCs may be induced to differentiate vs a neurogenic phenotype, in this case they do not appear to be functionally active [3]. Since their initial discovery, MSCs have been isolated from a variety of tissues including dental tissues and named human dental-derived mesenchymal stem cells (DSCs). Gronthos et al., first isolated a population of MSCs from dental pulp, with similar properties to BM-MSCs [4]. DSCs in teeth have been described and classified according to their origin tissue, in dental pulp stem cells (DPSCs), stem cells from human exfoliated deciduous teeth (SHEDs), periodontal ligament stem cells (PDLSCs), dental follicle stem cells (DFSCs), stem cells from apical papilla (SCAPs), and gingival MSCs (GMSCs) [4–9]. Notably, DSCs are derived from the neural crest, a transient population of cells derived from the ectoderm germ layer, the same one that gives rise to mature neurons, and for this reason they show more potent neurogenic capabilities compared to other MSCs [10]. Thus, DSCs, may be a good source of MSCs for the treatment of neurodegenerative disorders and neural regeneration thanks to their differentiation potential and paracrine effects, [11, 12]. Indeed, most pharmacological approaches to nervous system disorders are directed against symptoms of the pathology (e.g., neurodegenerative disorders, autoimmune diseases) [13]. Thus, their beneficial effects on patients are limited. For this reason, cell therapy (CT) is receiving increased attention as a possible alternative approach to such pathologies (Fig. 1). CT involves the application of autologous or allogeneic cells into a patient [14] in order to alleviate symptoms, or to regenerate a tissue damage. Strikingly, even the administration of material derived from SCs (i.e., secretome) has been proved beneficial in preclinical models of nervous system diseases.

In this review, we summarize the most recent research in the field of CT using human DSCs and their secretome. Highlighting analogies and differences between different types of DSCs, we recapitulate the main characteristics of these cells and the content of their secretome, as well as advances in materials to support and enable DSC growth. In the future, the increased use of neural crest SCs will provide invaluable therapeutic application and, ideally, the establishment of a biobank of these cells to perform autologous CT for the eradication of transplant rejection.

## Dental Stem Cells

DSCs have the advantages of being easily accessible by minimally invasive procedures [15], being expandable and maintaining relative genomic stability over a long period of time and exhibiting immunomodulatory properties [16]. Moreover, they are also able to differentiate toward the mesodermal lineage, but also show the ability to transdifferentiate into ectodermal and endodermal lineages [17]. For these reasons, DSCs have been considered a promising tool for therapeutic applications [18] due in part to their ability to secrete multiple factors essential for tissue regeneration and because they are obtained with fewer ethical or legal problems compared to other procedures [11, 19, 20]. All types of DSCs show different origins and characteristics, but at the same time, share similar expression profiles of surface markers as shown in Table 1. They express not only mesenchymal and embryonic SCs markers, but also neuronal markers, as they are derived from migrating neural crest cells, which originated from the germ layer of the embryonic ectoderm [10].

**Table 1:** Characteristics of different types of DSCs

DSCs	Origin	Characteristics	References
DPSCs	Isolated from dental pulp tissue of permanent teeth and are located predominantly in perivascular area of pulpal cavity	• They exhibit all the definitive stem cells ‘ properties, such as multi-differentiation and self-renewal potential • They maintain their stem cell properties even after cryopreservation • Positive markers: CD10, CD13, CD29, CD44, CD73, CD90, CD105, CD106, CD117, CD146, STRO-1 • Negative markers: CD14, CD19, CD24, CD34, CD45, HLA-DR • ESCs markers: OCT-4, Nanog, SSEA-1, SEEA-4, SOX-2 • Neural markers: β3-tubulin, NFM, Nestin, CNPase, S100, CD271	[4, 12, 21–27]
SHEDs	Isolated from pulp of exfoliated deciduous primary incisors of 7- to 8-year-old children	• They have typical stem cell properties including clonogenicity, cell proliferation and multipotency • They have higher proliferation rate and higher population doublings • They can differentiate into various cell types including neural cells, adipocytes, and odontoblasts • They can induce the formation of a bone-like matrix with a lamellar structure by recruiting host cells • They can remain undifferentiated and stable after long-term cryopreservation • Positive markers: CD13, CD29, CD44, CD73, CD90, CD105, CD106, CD146, CD166, STRO-1 • Negative markers: CD14, CD18, CD19, CD24, CD34, CD45 • ESCs markers: OCT-4, Nanog, SSEA-3, SSEA-4, NOTCH-1, OCT-4, SOX-2 • Neural markers: β3-tubulin, NFM, Nestin, CNPase, GAD, NeuN, GFAP, CD271, Vimentin, OCT- 4, PAX-6, NSE, MAP-2, PSA- NCAM	[5, 26, 28–31]
PDLSCs	Isolated from periodontal ligament (PDL), a connective tissue that connects the root of the tooth to the alveolar bone socket	• They exhibit a multipotent differentiation capacity • They are readily available, stable, and sufficient sources of cells that do not trigger immunological responses and have great features such as immune modulation and neuroprotection • Formation of PDL-cementum-like construction • Positive markers: CD13, CD29, CD44, CD49, CD73, CD90, CD105, CD146, CD166, CD271, CD10, STRO-1 • Negative markers: hematopoietic markers • ESCs markers: SSEA-1, SSEA-3, SSEA-4, TRA-1–60, TRA-1–81, OCT-4, Nanog, SOX-2, REX1, and ALP • Neural markers: Nestin, OCT-4, SSEA-4, CD271, SOX-10	[6, 32–35]
DFSCs	Isolated from the dental follicle, a connective tissue surrounding the impacted third molar tooth germ	• Formation of alveolar bone, Formation of PDL-cementum-like construction • They have better immunomodulatory and anti-apoptotic effects on the immune system than DPSCs and SHEDs • Positive markers: CD13, CD29, CD59, CD90, CD105, CD146, CD44, CD73, NOTCH-1, STRO-1 • Negative markers: hematopoietic markers • ESCs marker: OCT-4, Nanog, NOTCH-1, SOX-2 • Neural markers: OCT-4, SOX2, Nestin, SOX-2 • Show greater osteogenic properties (higher Runx2 and DSPP markers) than SHEDs and DPSCs	[9, 22, 36–39]
SCAPs	Isolated from the apical papilla, a soft tissue at the apices of developing permanent teeth	• Maintenance of root maturation • Formation of dentin-pulp-like complex • They can form cementum/PDL-like complex in vivo • They present self-renewal, proliferation, migration, differentiation, and immunosuppression, which support the application of SCAPs in stem cell-based therapy, including the immunotherapy and the regeneration of dental tissues, bone, neural, and vascular tissues • Positive markers: CD13, CD24, CD29, CD44, CD49, CD51, CD56, CD61, CD73, CD90, CD105, CD106, CD146, CD166, STRO-1, • Negative markers: CD14, CD18, CD34, CD45, CD117, CD150 • ESCs marker: OCT-4, Nanog, NOTCH-1, SOX-2 • Neural markers: OCT-4, SOX2, Nestin	[16, 36, 37, 40–43]
GMSCs	Isolated from healthy gingival tissues	• Easy to isolate, long-term stability • They exerted anti-proliferative and pro-apoptotic effects on oral cancer cells both in vitro and in vivo • Positive markers: CD13, CD29, CD44, CD73, CD90, CD105, CD146, STRO-1 • Negative markers: CD34, CD45 • ESCs marker: SSEA-4, OCT-4, Nanog • Neural markers: Nestin, SOX10, β3-tubulin, NFM, CNPase	[37, 44–47]

## Maintenance and Survival of DSCs

### Differentiation Capabilities and Maintenance of DPSCs’stemness

DPSCs have a high differentiating capacity. In fact, it has been demonstrated that they can differentiate into different cell lineages including endodermal, mesodermal, and ectodermal lineages, respectively [19, 23, 48–59]. Furthermore, with the identification of functional markers and appropriate culture conditions for SC selection, it will be possible to target DPSCs for clinical applications [60]. In this regard, several authors have demonstrated that there are biological factors (such as tooth type, age, genetic background and lifestyle) that should be considered before tooth selection, and have compared traditional isolation, culture and storage techniques with improved methods or protocols such as the use of a serum/xenon-free culture medium to prolong the stemness of DPSCs [61, 62]. Diomede et al., studied the expression of proteins involved in cell proliferation/senescence and embryonic stem cell markers during early and late passages in MSCs obtained from dental pulp tissues, suggesting that the presence of embryonic and proliferation markers in late passages could potentially support the application of DSCs in clinical trials based on stem cell therapy [63].

### Differentiation Capabilities and Maintenance of SHEDs’stemness

SHEDs also have a high differentiation capacity [5, 64–68]. SHEDs have a greater self-renewal potential than DPSCs even when subjected to adverse culture conditions [69, 70]. SHEDs, in addition to expressing the neural crest marker just like DPSCs [65], also express a wide range of lineage-specific markers and genes as shown in Table 1 [71, 72]. During long-term culture, SHEDs do not undergo spontaneous degeneration or differentiation [73]. Furthermore, SHEDs have been shown to possess higher proliferation rates and differentiation potential after cryopreservation and long-term storage (for two years) than DPSCs [29, 74] and retain similar properties to those obtained from fresh tissue [70, 75] that can be used for cell-based therapy [76]. It has been shown that the cell culture microenvironment can also influence the secretion and differentiation potential of pulp cells [77]. SHEDs and DPSCs, despite being SCs originating from the same tissue source, but from two different time points, show differences in their differentiation capacity towards major lineages [5, 65, 78, 79]. Naz et al., demonstrated that SHEDs have a better proliferation and self-renewal and better osteogenic differentiation capacity compared to DPSCs [80, 81]. Kanafi et al., further demonstrated that SHEDs have greater differentiation potential towards insulin-producing cells than DPSCs in the presence of appropriate inductive signals [70]. DPSCs and SHEDs are capable of regenerating pulp and dentin and therefore have the potential to be used as a source of pluripotent stem cells for future cell therapies in medicine and dentistry [78, 82, 83].

### Differentiation Capabilities of Other DSC Types

PDLSCs and SCAPs possess the same in vitro multilineage differentiation potential like that of DPSCs and SHEDs [8, 34, 35, 40, 43, 84], making them good candidates in regenerative medicine to promote both dental and non-dental tissue regeneration [84]. DFSCs can differentiate into cementoblasts in vivo [85] and they can also be directed to differentiate into conventional multidirectional lineages [8, 86]. Instead, GMSCs can be targeted for osteogenic, adipogenic, chondrogenic, neurogenic, endothelial-like, odontogenic, and myogenic differentiation [46, 47].

## Development of 3D Models to Study Neurological Disorders

Conventional in vitro models for studying SCs differentiation are usually cultured in two dimensions (2D). The in vitro three-dimensional (3D) model, which should ideally mimic the SCs microenvironment in vivo, is potentially useful for inducing stem cell-derived tissue formation. Biodegradable scaffolds play an important role in creating 3D structures to guide tissue formation. Furthermore, the diffusion of nutrients, oxygen, and bioactive factors through the 3D constructs shows increased efficiency for cell survival over long periods of time. Ideally, the scaffold should have mechanical properties that are consistent with the anatomical site where it is to be implanted. Recently, progress has been made in the development of natural and synthetic biomaterials for peripheral nerve (PN) repair to address the challenging clinical problem of damaged PN regeneration. These materials can be used as membranes useful in the repair of nerves and nerve ducts. For example, Wang et al., in 2020 demonstrated that collagen scaffold can promote the recovery of the functional facial nerve [87]. Moreover, the use of Silymarin nanoparticles loaded into the chitosan conduit was shown to improve functional recovery of transected sciatic nerve in rats [88]. On the same topic, Zorba Yildiz et al., investigated the preparation of a biohybrid hydrogel bioink containing graphene for use in peripheral tissue engineering and demonstrated that using of this bioink induced the neural differentiation of SCs [89]. Moreover, Su and Pan demonstrated the possibility to induce SHEDs’ neural differentiation by using 3D polydimethylsiloxane (PDMS) scaffolds in the perfusion system operating in the high-concentration of a rat Schwann cell (RSC)-seeded culture medium [90]. Other authors demonstrated that engineered Elastin-like proteins can support regeneration of PN [91]. The main scaffolds utilized to support cells involved in the repair process such as neurons, Schwann cells, macrophages, and blood vessels can be divided into biomaterials scaffold and biological scaffold. Both synthetic (biomaterials scaffold) and natural (biological scaffold) material show several tangible advantages and disadvantages attributed to both options [92, 93]. Due to their architectural structure, both types of scaffolds mimic the native extracellular matrix (ECM). The main structural difference is that biomaterial scaffolds are made of synthetic polymers or purified natural polymers, whereas biological scaffolds are composed of decellularized mammalian tissue [94].

### Structural Properties (Characteristics) of Scaffolds

In general, biomaterials scaffolds are considered better than biological scaffolds for several characteristics such as precise geometrical pattern, biocompatibility, porosity, and stiffness to obtain a more easily and precise architecture and they can be more easily adapted, to improve cell adhesion and tune mechanical properties [95–97]. However, natural materials possess innate cell binding motifs, produce harmless degradation products, and effectively mimic physiological-like tissue remodeling and activate repair pathways [98, 99].

#### Geometrical Pattern

Geometrical pattern is one of the features able to modulate SCs differentiation. As reported by Das and Bellare also DPSCs in Customized 3D Nanofibrous Scaffolds for regeneration of Peripheral Nervous System (PNS) can support DPSCs proliferation and their subsequent neural differentiation [100]. Together with other studies, all authors demonstrated that transplanted scaffold containing DPSCs may provide a promising strategy for neuron repair, functional recovery, and neural tissue regeneration [101, 102].

#### Stiffness

Stiffness is one of the characteristics that can affect in a cell-specific way cells behavior during regeneration. For example, Sridharan et al., demonstrated that upon implantation of a biomaterial, MSCs and macrophages both contribute to the regeneration cascade of events [103]. Besides, Srinivanan et al., demonstrated that stiffer substrates can modulate human neural crest derived stem cell differentiation via CD44 mediated signaling [104]. Other studies reported that Schwann cells can develop normally on both stiff and soft hydrogels but activate different intracellular pathways in response to different substrate stiffness [105]. On this topic, Liu et al., demonstrated that exosome-loaded hydrogel stiffness plays an important role in tissue regeneration by regulating exosome release behavior [106]. Also, Guo et al., fabricated highly vascularized scaffolds embedded with human DPSCs, that thanks to their paracrine-mediated angiogenic and neuro-regenerative potentials were capable of augmenting and modulating Spinal Cord Injurie (SCI) repair [107].

#### Porosity

Also, porosity is an important parameter affecting neural cell regeneration. For example, nanostructures surface topographies have been developed to mimic microstructures for neuronal axons and Schwann cells also incorporating matrix proteins or peptides [108]. Injectable hydrogel systems have been quite extensively studied to deliver therapeutic molecules in various neurological disorders. This system has been mainly developed for the treatment of tumors to maintain a therapeutic concentration of the drug for a prolonged time in the local area and so to avoid toxicity [109]. Also, hydrogels composite comprising of dextran dialdehyde and gelatin, loaded with dopamine have been used in animal models of Parkinson’s disease (PD) to release neurotransmitters [94]. Hydrogel matrices have also been used for embedding different neurotrophic factors by an affinity-based system. The negatively charged sulfate groups present in heparin were capable of immobilizing neurotrophic growth factors such as nerve growth factor (NGF), glial cell derived neurotrophic factor (GDNF) and Neurotrophin 3 (NT-3) that could be released in the local area of treatment [110–112]. Ansari et al., demonstrated that PDLSCs and GMSCs promote nerve regeneration when encapsulated in a 3D scaffold based on alginate and hyaluronic acid hydrogels that, due to its elasticity, was capable to sustain the release of NGF [113]. Ueda et al., demonstrated that chitosan scaffolds combined with Basic fibroblast growth factor (bFGF) facilitated the neural differentiation of DPSCs [114].

#### Viscosity

Viscous characteristics together with elastic properties are also important in brain tissues that in a process of “stress-relaxation” responds to mechanical perturbations [115]. For example, hydrogel matrix allows to reproduce several factors that are required for the axonal growth such as mechanical stress experienced by the neural membrane, neuronal cytoskeletal dynamics, and mechano-sensing ability of the neurons. Luo et al., developed a 10% GelMA hydrogel mix with recombinant human bFGF and DPSCs to fill a cellulose/soy protein isolate composite membrane (CSM) tube. They constructed a nerve-like conduit, demonstrating that CSM-GFD could be a promising tissue engineering approach to replace the conventional nerve autograft to treat the large gap defect in peripheral nerve injuries [116]. Similarly, Li et al., demonstrated that dental follicle cells (DFCs) seeded on Aligned electrospun PCL/PLGA material (AEM) could stretch along the oriented fibers and proliferate efficiently and transplanted in rats contributed to restore the defect in rat spinal cord [117]. One of the goals of tissue engineering is therefore to try to exploit the innate regenerative capacity of SCs. Designing the scaffold in such a way that the scaffold itself delivers regenerative signals to cells could be a good model. In fact, in addition to the biomechanical contribution, cellular behavior is strongly influenced by biological and biochemical signals coming from the ECM.

#### Conductivity

Another property able to influence the cellular behaviour in response to a regenerative stimulus could be electrical conductivity of scaffolds. Liu et al., demonstrated that a 3D electroconductive gelatin methacryloyl-multiwalled carbon nanotube/cobalt3D conductive hydrogel scaffolds accelerated the neural differentiation of SCAPs. Considering these aspects, the novel electroconductive GelMA-MWCNTs/Co hydrogel synergized with ES greatly promotes SCAPs neuronal differentiation [118]. Therefore, the use of scaffolds as delivery systems for growth factors, adhesion molecules and cytokines has recently received attention [119]. This condition would be particularly interesting for stem cell models such as DSCs. As reported so far, the combined application of technologies such as scaffolding materials and SCs is thought to have enormous potential for tissue regeneration [18].

## DSCs Secretome

Currently, research is focusing on the therapeutic applications of DSCs, particularly in preventing and treating neurological diseases and nerve injuries [37]. The role played by these cells in the reparative process is mainly attributed to paracrine mechanisms mediated by their secreted factors [53]. Gnecchi et al., demonstrated that MSCs mediate their therapeutic effects through the secretion and release of trophic molecules known as “secretome”. This evidence allowed the development of the theory that the therapeutic applicability of MSCs in regenerative medicine is based on their ability to homing to the site of tissue injury and differentiate into different functional cell types, leading to tissue repair [120]. Interestingly, some studies also suggested the superiority of the secretome obtained from DSCs compared to other MSCs sources, such as BM-MSCs and adipose-derived mesenchymal stem cells (AD-MSCs), for neuroprotection [121]. The secreted factors can be found in the cell culture medium called Conditioned Medium (CM) and in the Exosomes (Exos) they secrete [122], representing an attractive, noninvasive, and acellular tool for therapeutic approaches against various disorders [123]. A summary of the main factors found in the DSCs’ secretome is given in Table 2.

**Table 2:** Main factors found in DSCs’ secretome

DSCs	Secretome	Contained factors	References
DPSCs	CM	Ang-2, EGF, Endoglin, Endothelin-1, Eotaxin-1, FGF-1, FGF-2, Flt-3L, Follistatin, G-CSF, GM-CSF, GRO pan, HB-EGF, HGF, IFNα2, IFNγ, IL-12(p40), IL-12(p70), IL-13, IL-15, IL-1B, IL-5, IL-8, IL-9, IP-10, Leptin, MCP-1, MCP-3, PDGF-AA, PDGF-BB, PLGF, RANTES, TGF-α, TGF-β1, TGF-β2, TGF-β3, TNFα, TNFβ, VEGF-A, VEGF-C, VEGF-D, IGF-1, IL10, IGFBP-6, NT-3, BMP-4, MIP-1δ, NAP-2, TGF-β3, TGF-β1, MIP-3α, TNF-α, TNF-β, ICAM-1, NT-4, I-TAC, TARC, Axl, THPO, TECK, Acrp-30, ICAM-3, EGFR, AgRP, XCL-1, MIF	[124, 125]
SHEDs	CM	FGF-2, IL-10, PDGF, SDF-1, Ang-1, TGF-β3, HGF, INF-γ, VEGF, and IL-6	[126]
PDLSCs	CM and Exos	99 proteins, including matrix proteins, enzymes, growth factors, cytokines, and angiogenic factors, IL-10, SDF-1α, TGF-β, IL-15, NT3, MCP-1 and MIP-1α	[127–132]
SCAPs	CM	2046 proteins, included chemokines, angiogenic, immunomodulatory, antiapoptotic, and neuroprotective factors, ECM proteins	[133]
GMSCs	CM	NGF, NT3, IL-10, and TGF-β	[134]

### Conditioned Media and Exosomes in Neurological Disorders

#### DPSCs

It is important to note that donor age and in vitro microenvironmental conditions may also influence secretome composition. Indeed, DPSC-CM obtained under normoxic conditions was reported to be enriched in molecules with anti-inflammatory, tissue repair, and regenerative properties compared to CM obtained under hypoxic conditions [135]. Confirming that hypoxia appears to promote neuronal differentiation of DPSCs, Delle Monache et al. demonstrated that administration of DPSC-CM under hypoxic conditions resulted in neuronal differentiation of both SH-SY5Y neuroblastoma cells and undifferentiated DPSCs [23]. DPSC-CM promoted neurite outgrowth in dorsal root ganglion (DRG) neurons. In particular, the total length and joint number of neurites increased after CM treatment. Furthermore, DPSC-CM promotes Schwann cell viability and myelin formation [136]. DPSCs’ secretome also showed beneficial effects in models of neurodegenerative diseases. Ahmed et al., described that treatment with DPSCs’secretome reduced amyloid β (Aβ) cytotoxicity in an in vitro model of Alzheimer’s disease (AD), increasing cell viability and reducing apoptosis [137]. Wang et al., demonstrated for the first time the therapeutic benefits of DPSC-CM in amyotrophic lateral sclerosis (ALS) with beneficial effects on direct motor neuron protection and lifespan prolongation [138]. The protective effect of DPSC-CM has also been demonstrated in the treatment of experimental autoimmune encephalomyelitis (EAE), the main murine model of multiple sclerosis (MS). Shimojima et al., showed that DPSC-CM administration reduced the expression of inflammatory cytokines in the spinal cord, inhibited demyelination, and improved clinical scores of EAE [139]. DPSC-Exos may be more suitable for the treatment of neurodegenerative diseases than MSCs derived from mesodermal tissues [138]. They may be an effective drug carrier for the treatment of various diseases, especially neurological disorders such as PD [140, 141].

#### SHEDs

The SHED-CM strongly promoted the expression of anti-inflammatory cytokines, angiogenic and anti-apoptotic factors [142], making this a potential therapeutic tool in neurological disorders. Different studies reported beneficial effects of SHED-CM both in vitro and in vivo PD models [143]. Mita et al., demonstrated that SHED-CM attenuated pro-inflammatory responses induced by β-amyloid plaques and generated an anti-inflammatory/tissue regeneration environment, which was accompanied by the induction of M2-like anti-inflammatory microglia, suggesting that SHED-CM can provide significant therapeutic benefits for AD [144]. Matsubara et al., demonstrated that SHED-CM, administered intrathecally into injured spinal cord in the rat during the acute postinjury period, caused a remarkable functional recovery related to the induction of anti-inflammatory M2 macrophage [145]. Miura-Yura et al., described that SHED-CM significantly promoted neurite outgrowth of dorsal root ganglion neurons compared to basal DMEM indicating that SHED-CM might have a therapeutic effect on diabetic polyneuropathy through the promotion of neurite outgrowth, and the increase in capillaries may contribute to the improvement of neural function [146]. In an animal model of superior laryngeal nerve injury, the systemic administration of SHED-CM induced functional recovery, increasing the degree of myelination, and promoted axonal regeneration shifting macrophages toward the M2 phenotype [147]. Li et al., injected SHED-Exos into a traumatic brain injury (TBI) rat model and observed that SHED-Exos contributed to rat motor functional restoration and cortical lesion reduction by shifting microglia polarization [148]. Narbute et al., showed that SHED-Exos significantly improved the gait impairments and contralateral rotations in the unilateral 6-hydroxydopamine (6-OHDA) rat model of PD [149]. In terms of anti-inflammatory effect, SHED-Exos significantly suppressed the carrageenan-induced acute inflammation in vivo [150]. Similarly, Luo et al., showed that SHED-Exos markedly reduced the inflammation in chondrocytes derived from the temporomandibular joint through delivering miR-100-5p [151].

#### PDLSCs

PDLSCs secretome can reduce oxidative stress and inflammation in injured neurons and can increase the functionality of the PI3K/Akt/mTOR axis which results in restoring BDNF production. Moreover, the CM has a neuroprotective effect due to containing NT-3, and IL‐10, and the presence of growth factors and immunomodulatory cytokines [152]. PDLSC-CM is useful in enhancing long-term neuronal regeneration in spinal cord injury [153]. Rajan et al., showed that PDLSCs-CM obtained from patients with relapsing‐remitting MS (RR-MS) showed anti-inflammatory and antiapoptotic effects when injected in a mouse model of MS [129]. Interestingly, CM obtained from PDLSCs cultured under hypoxic conditions was efficacious in ameliorating clinical and histological disease scores in EAE mice. This treatment reduced inflammatory cell infiltration and increased remyelination in the spinal cord.

#### SCAPs

Yu et al., profiled the secretome of human SCAPs by comparing it to that of BM-MSCs. A total of 2,046 proteins were detected in the SCAP-CM. Chemokines were included as well as angiogenic, immunomodulatory, anti-apoptotic and neuroprotective factors and ECM proteins [133]. In another study, Yu et al., compared the osteo/odontogenic, angiogenic, and neurogenic effects of soluble factors released from SCAPs and BM-MSCs in vitro on the proliferation and differentiation of dental pulp cells (DPCs). They demonstrated that CM released from SCAPs had a greater osteo/odontogenic and neurogenic inductive effect on DPCs than BM-MSCs-CM. This indicates that SCAPs-CM may serve as an additive to enhance pulp tissue repair and regeneration [154].

#### GMSCs

Rajan et al., demonstrated the presence of NGF, NT-3, IL-10 and TGF-β in GMSC-CM, which provide neuroprotection in scratch-damaged motor-neuron-like NSC-34 cells suggesting a potential therapeutic application of GMSC-CM in motor neuron degenerative diseases [134]. Although few studies are currently available on the potential therapeutic applications of GMSC-Exos, they hold a great promise for tissue regeneration. Jiang and Xu demonstrated that GMSC-Exos facilitated the osteogenic differentiation of MC3T3-E1 cells [155]. In a high-lipid microenvironment, GMSC-Exos suppressed lipid accumulation, transformed pro-inflammatory macrophages to an anti-inflammatory phenotype, and decrease the secretion and expression of inflammatory factors including IL-6, IL-1β, TNF-α, and cluster of differentiation [156]. Rao et al., showed that GMSC-Exos enhanced the proliferation of Schwann cells and the growth of the dorsal root ganglion neuron axon as well as promoting the formation of nerve fibers and myelin, which subsequently contributed to the recovery of motor skills, nerve conduction function, and muscle movement [157].

## Conclusion

DSCs, have been shown to possess a remarkable neuroregenerative potential due to their neural crest origin. Not only the cells but also their secretome exhibit the same enhanced neuroprotective and neuroregenerative properties. The studies evaluated in this review have shown that both CM and Exos contain neurotrophins and molecules with neuroprotective action, even at higher levels than other MSCs. DSC-CM and DSC-Exos stimulated neurite outgrowth and exhibited neuroprotective effects in preclinical models of neurological disease and neuronal injury. DSC-CM represent an attractive, non-invasive, and acellular tool for therapeutic approaches against various disorders. DSC-Exos possesses unique advantages such as high drug loading capacity, high specificity, low immunogenicity, excellent biocompatibility, ease of obtaining, low side effects and nanometer size. DSC-Exos is emerging as a promising and practical therapeutic approach for tissues repair and regeneration. Currently, DPSC and SHED secretomes are the most studied. However, several studies have also highlighted the neuroprotective effects of PDLSC and GMSC secretomes. Interestingly, some studies have also suggested the superiority of DSCs derived secretome over other MSCs sources, such as BM-MSCs and AD-MSCs, for neuroprotection. Furthermore, the mechanical properties of the substrate to which the cells are attached are fundamental fin regulating cellular mechano-transduction and the subsequent cellular behavior, especially when further technologies allow the substrate itself to become biologically active. This has important implications for development, differentiation, disease, and regeneration. The proliferation, paracrine effect, and multidirectional differentiation potential of DSCs support the application of DSCs in regenerative medicine [18]. In conclusion, DSC secretomes in combination or not with biomaterials and biological scaffold are emerging as a promising and practical therapeutic approach for the repair and regeneration of different tissues, especially in the neuroregenerative field. It is thought to be useful for the development of new neuroprotective therapies.

## Data Availability

Not applicable.

## Abbreviations

SCs: Stem cells
MSCs: Mesenchymal stem cells
DSCs: Dental-derived MSCs
DPSCs: Dental pulp stem cells
SHEDs: Stem cells from human exfoliated deciduous teeth
PDLSCs: Periodontal ligament stem cells
DFSCs: Dental follicle stem cells
SCAPs: Stem cells from apical papilla
GMSCs: Gingival MSCs
CM: Conditioned medium
Exos: Exosomes
